# Responses of *Coix lacryma-jobi* L. to Exogenous Phenolic Acid Treatments: Effects on Growth, Antioxidant Responses, and Leaf Metabolome

**DOI:** 10.3390/plants15132015

**Published:** 2026-06-29

**Authors:** Yihang Liu, Qi Miao, Muhammad Riaz, Xianyong Lu, Yujiao Wang, Yi Zhou, Ping Zhang, Yulong Li, Yongle Wang, Jiabao Zhu, Hao Xia

**Affiliations:** 1 Industrial Crop Institute, Anhui Academy of Agricultural Sciences (AAAS), Hefei 230001, China; liuyihang0208@163.com (Y.L.); nzg1021@163.com (X.L.); wangyujiao0207@163.com (Y.W.); w18714905868@163.com (Y.W.); 2 College of Resources and Environment, Anhui Science and Technology University, Chuzhou 233100, China; zhouy@ahstu.edu.cn (Y.Z.); zhangp@ahstu.edu.cn (P.Z.); 3 Anhui Provincial Key Laboratory of Nutrient Cycling and Arable Land Conservation, Soil and Fertilizer Institute, Anhui Academy of Agricultural Sciences (AAAS), Hefei 230001, China; miaoqibody@163.com; 4 Microelement Research Center, College of Resources and Environment, Huazhong Agricultural University, Wuhan 430070, China; riaz1480@hotmail.com; 5 Institute of Crop Research, Anhui Academy of Agricultural Sciences (AAAS), Hefei 230001, China; 13655178998@163.com

**Keywords:** *Coix*, phenolic acid, differential metabolites, continuous cropping obstacles, amino acid

## Abstract

Phenolic acids are known allelopathic compounds that may serve as the primary cause of continuous cropping obstacles in *Coix lacryma-jobi* L. (*Coix*). However, the concentrations, types, and specific stress responses of *Coix* to these phenolic acids still require further investigation. In this study, the cultivar ‘Wanyi 2′ was used to examine the effects of different phenolic acids and their concentrations on the growth of *Coix*. Four concentrations (0 mg/L, 10 mg/L, 100 mg/L, and 1000 mg/L) and four phenolic acid types (p-hydroxybenzoic acid, salicylic acid, cinnamic acid, and ferulic acid) were used to assess their influences on plant growth, leaf physiological parameters, and metabolic pathways under greenhouse conditions. In this greenhouse pot experiment, the effects of the four phenolic acids showed a similar tendency: a low concentration (10 mg/L) tended to promote the growth and root development of *Coix* seedlings, whereas high concentrations (100 and 1000 mg/L) generally showed inhibitory effects. Among these phenolic acids, ferulic acid exhibited the strongest inhibitory effect at the highest concentration (1000 mg/L), while salicylic acid showed the most pronounced growth-promoting effect at low concentrations (10 mg/L). In addition, high levels of phenolic acids markedly increased antioxidant enzyme activities and oxidative stress-related substances in *Coix* leaves, while reducing soluble sugar (SS) and soluble protein (SP) contents. Our data suggest that under phenolic acid stress, *Coix* leaves exhibited changes in the metabolism of phenolic acids (e.g., 4-methoxysalicylic acid, gallic acid) and amino acids (e.g., glutathione, proline), which may be associated with the adaptive response to allelochemical-induced stress. Overall, this study provides insights that may support strategies to optimize plant growth regulators and mitigate continuous cropping barriers in *Coix*.

## 1. Introduction

*Coix lacryma-jobi* L. (*Coix*) is an annual plant belonging to the Poaceae family and possesses high nutritional and medicinal value [1]. In recent years, continuous cropping obstacles have become increasingly prominent [2]. Continuous cropping obstacles have adversely affected the healthy and sustainable development of the *Coix* industry [3]. After six years of cultivation, the yield of *Coix* decreased by 44.13–53.43%, accompanied by decreases in soluble sugars, lysine, and starch [4]. Therefore, investigating the concentration-dependent effects of phenolic acids on *Coix* growth and physiology is an important step toward understanding the potential role of these phenolic acids in continuous cropping obstacles.

Continuous cropping can lead to stunted growth, abnormal root development, reduced stress resistance, severe soil-borne diseases and pests, and a decline in yield and quality [5]. In severe cases, this phenomenon may even result in plant death [6,7]. Allelopathic substances produced under long-term monoculture can gradually reduce enzyme activities in leaves, while increasing malondialdehyde [8]. In addition, continuous cropping hinders root elongation, root vitality, and biomass, and consequently limits root distribution, nutrient uptake, and water absorption [9,10].

Soil degradation (including nutrient imbalance and microbial community disorder) and allelopathic autotoxicity are considered the main causes [11,12,13]. The self-toxic effects of allelochemicals are a key contributing factor, as they can inhibit the growth of the same crop species and increase disease incidence [14,15]. The accumulation of allelochemicals significantly reduces crop yield and quality [16]. For example, p-coumaric acid may inhibit the growth of tobacco plants by directly affecting their physiological processes and indirectly disturbing the microbial ecological balance in soil [17]. Under long-term continuous cropping, the biosynthesis and secretion of triterpenoid saponins in the rhizosphere increase, causing accumulation of autotoxic substances and depletion of prebiotics, and thereby inhibiting alfalfa growth [18]. Phenolic acids are regarded as crucial allelopathic autotoxic substances due to their high biological activity and abundance in soil systems [19]. Therefore, investigating the effects of phenolic acids on the autotoxicity of *Coix* is of substantial importance for alleviating continuous cropping obstacles.

Currently, research on continuous cropping obstacles has extensively focused on other field crops (e.g., peanut, strawberry, tobacco, cucumber) and medicinal plants (e.g., *Panax notoginseng*, *Rehmannia glutinosa*), where specific phenolic acids have been identified as key autotoxins. However, systematic analyses of the mechanisms underlying continuous cropping obstacles remain limited in *Coix*, a crop of high nutritional and medicinal value that suffers severe yield declines under prolonged monoculture. In particular, the concentration-dependent effects of phenolic acids on *Coix* growth, root development, and leaf metabolic functions have not been investigated. Unlike most previous studies that focused on single phenolic acids or pairwise comparisons in other crops, our study systematically compares four major phenolic acids under the same experimental conditions. Therefore, this study investigates phenolic acid effects on the growth of *Coix* under controlled greenhouse conditions. By examining the effects of these treatments on root development, leaf physiological parameters, and metabolomic profiles, the following objectives are addressed: (1) To investigate the effects and mechanisms of different concentrations of phenolic acids on the growth, physiology, and metabolism of *Coix*; (2) To compare the differences in growth of *Coix* under various types of phenolic acids.

## 2. Results

### 2.1. Effects of Different Concentrations and Types of Phenolic Acids on Agronomic Traits of Coix Seedlings

As shown in Table 1, different types and concentrations of phenolic acids significantly affected the growth and development of *Coix*. Compared with the CK treatment, fresh biomass, dry biomass, and plant height were significantly increased under low phenolic acid concentrations (10 mg/L), whereas high concentrations (100 mg/L and 1000 mg/L) markedly inhibited *Coix* growth and development (Table 1). Relative to the CK treatment, under the low concentration (10 mg/L) of each phenolic acid, the increases across the four phenolic acids ranged from 19.54 to 59.68% for stem fresh weight, from 11.20 to 60.25% for root fresh weight, from 42.98 to 118.18% for stem dry weight, from 32.35 to 72.05% for root dry weight, and from 1.90 to 21.08% for plant height (Table 1). However, at the highest concentration (1000 mg/L) of each phenolic acid, the decreases across the four phenolic acids ranged from 50.95 to 60.90% for leaf fresh weight, from 35.21 to 72.24% for stem fresh weight, from 18.57 to 60.71% for root fresh weight, from 62.86 to 79.29% for leaf dry weight, from 52.07 to 76.86% for stem dry weight, from 23.53 to 50.00% for root dry weight, from 19.91 to 41.27% for plant height, from 16.99 to 41.11% for stem diameter, and from 2.70 to 15.96% for SPAD (Table 1). Among the four phenolic acids tested, ferulic acid exhibited the strongest inhibitory effect on *Coix*. According to the two-way ANOVA results, both the main effects and the interaction effects of phenolic acid type and concentration significantly influenced the agronomic traits of *Coix* (Table 1).

The tolerance index of the aboveground parts (TIA) and underground parts (TIU) decreased significantly with increasing phenolic acid concentration (Table S1). Compared with low phenolic acid concentrations (10 mg/L), TIA and TIU were reduced by 74.81–84.51% and 47.26–69.71%, respectively, under high concentrations (1000 mg/L) (Table S1). In addition, TIA and TIU were significantly affected by high concentrations of different phenolic acids (100 and 1000 mg/L). Among all phenolic acid treatments, ferulic acid resulted in the lowest TIA and TIU values (Table S1).

### 2.2. Effects of Different Concentrations and Types of Phenolic Acids on Root Development of Coix Seedlings

The growth and development of roots were influenced to varying degrees by the types and concentrations of phenolic acids (Table 2). Compared with the CK treatment, root growth indicators were maximally enhanced at 10 mg/L, whereas significant inhibition of root growth and development was observed at 1000 mg/L (Table 2). Relative to CK, under the low concentration (10 mg/L) of each phenolic acid, the increases across the four phenolic acids ranged from 11.73 to 80.64% for root length, from 9.18 to 75.72% for surface area, from 23.24 to 62.66% for root volume, and from 28.06 to 244.87% for root tips (Table 2). In contrast, at 1000 mg/L, the decreases across the four phenolic acids ranged from 15.17% to 39.88% for root surface area, from 25.00% to 35.42% for average diameter, and from 13.84% to 55.63% for root volume (Table 2). Among all phenolic acid treatments, root growth showed the strongest inhibition under low concentrations of ferulic acid, whereas at high concentrations, p-hydroxybenzoic acid exerted the most pronounced inhibitory effect (Table 2). In addition, with increasing phenolic acid concentrations, root length ratio (RLR), root fineness (RF), and root mass ratio (RMR) exhibited a significant upward trend. Compared with CK, the increases across the four phenolic acids (at 1000 mg/L) ranged from 50.92% to 92.25% for RLR, from 52.40% to 83.98% for RF, and from 33.33% to 85.71% for RMR (Table S1). Based on the two-way ANOVA, both phenolic acid type and concentration, as well as their interaction, significantly affected root development parameters (length, surface area, average diameter, volume, and tips) (Table S1).

### 2.3. Effects of Different Concentrations and Types of Phenolic Acids on Antioxidant Enzymes and Antioxidant Substances of Coix Seedlings

Different concentrations and types of phenolic acids caused significant changes in the antioxidant enzyme activities and antioxidant substances in *Coix* leaves (Table 3). Compared with the CK treatment, the activities of SOD, POD, and CAT increased by 7.39–134.17%, 8.09–29.54%, and 34.15–104.88%, respectively, under high phenolic acid concentrations (1000 mg/L) (Table 3). Across the four phenolic acid types, when applied at the highest concentration (1000 mg/L), the contents of H_2_O_2_, O_2_^−^, MDA, and Pro in *Coix* leaves increased compared with their respective CK treatments (Table 3). The overall ranges of increase were 12.62–25.23% for H_2_O_2_, 46.58–67.55% for O_2_^−^, 32.46–98.79% for MDA, and 39.07–56.27% for Pro (Table 3). Conversely, at 1000 mg/L, SS and SP contents decreased across the four phenolic acids, with overall ranges of 1.48–10.39% and 37.19–63.64%, respectively (Table 3). Overall, two-way analysis of variance revealed that the main effects of phenolic acid type and concentration, as well as their interaction effect, exerted significant influences on the activities of antioxidant enzymes and antioxidant substances (except for Pro) in leaves.

### 2.4. Effects of Different Concentrations and Types of Phenolic Acids on Leaf Metabolites of Coix Seedlings

Under most phenolic acid treatments, the number of significantly downregulated metabolites exceeded the number of upregulated ones (Figure S1). Compared with CK, significant decreases were observed in 82 (T1), 150 (T3), 122 (T4), 119 (T6), 119 (T7), 122 (T9), 168 (T10), and 167 (T12) metabolites, while significant increases were detected in 56 (T1), 100 (T3), 102 (T4), 166 (T6), 104 (T7), 102 (T9), 116 (T10), and 167 (T12) metabolites (Figure S1). These differential metabolites (Ala-Phe-Arg, oxolinic acid, salicylic acid, etc.) were primarily involved in amino acid biosynthesis, cofactor biosynthesis, and methionine metabolism according to enrichment analysis (Figures S2 and S3).

**Figure 1:**
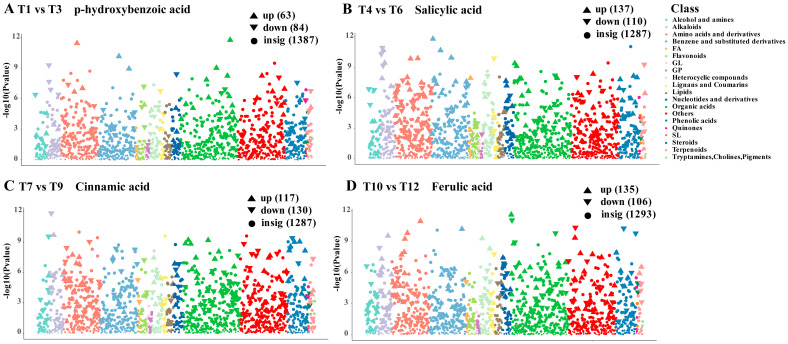
The scatter diagram of differential substance classification between low (10 mg/L: T1, T4, T7, T10) and high (1000 mg/L: T3, T6, T9, T12) concentration groups. (**A**): T1 vs. T3 (p-hydroxybenzoic acid); (**B**): T4 vs. T6 (salicylic acid); (**C**): T7 vs. T9 (cinnamic acid); (**D**): T10 vs. T12 (ferulic acid).

**Figure 2:**
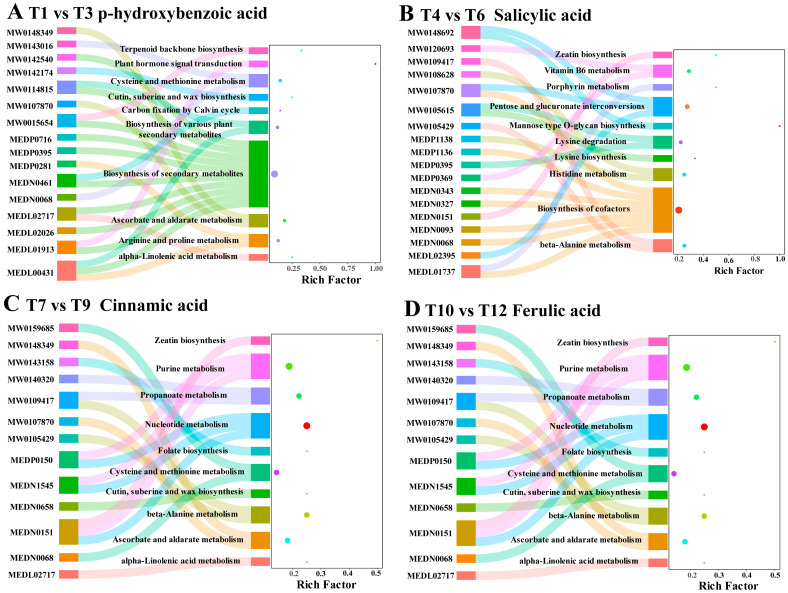
The Sankey and bubble plot of KEGG (top 10 pathways) and dynamic distribution map of differences in metabolites between low (10 mg/L: T1, T4, T7, T10) and high (1000 mg/L: T3, T6, T9, T12) concentration groups. (**A**): T1 vs. T3 (p-hydroxybenzoic acid); (**B**): T4 vs. T6 (salicylic acid); (**C**): T7 vs. T9 (cinnamic acid); (**D**): T10 vs. T12 (ferulic acid).

Compared with low phenolic acid concentrations (10 mg/L), the number of significantly downregulated metabolites was 84 (p-hydroxybenzoic acid), 110 (salicylic acid), 130 (cinnamic acid), and 106 (ferulic acid), whereas the number of significantly upregulated metabolites was 63, 137, 117, and 135, respectively, under high phenolic acid concentrations (1000 mg/L) (Figure 1), which were involved in the biosynthesis of secondary metabolites, nucleotide metabolism, purine metabolism, and beta-alanine metabolism (Figure 2).

### 2.5. Metabolic Pathways of Phenolic Acids and Amino Acids in Leaves of Coix L Under Phenolic Acid Stress

**Figure 3:**
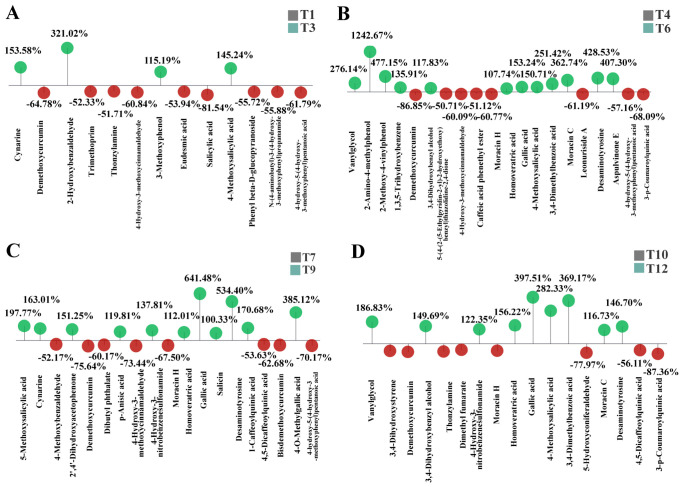
Analysis of phenolic acid between low (10 mg/L: T1, T4, T7, T10) and high (1000 mg/L: T3, T6, T9, T12) concentration groups. (**A**) T1 vs. T3 (p-hydroxybenzoic acid); (**B**) T4 vs. T6 (salicylic acid); (**C**) T7 vs. T9 (cinnamic acid); (**D**) T10 vs. T12 (ferulic acid). Green: Compared with low-concentration treatments, the contents of phenolic acids in leaves increased under high-concentration treatments; Red: Compared with low-concentration treatments, the contents of phenolic acids in leaves decreased under high-concentration treatments.

**Figure 4:**
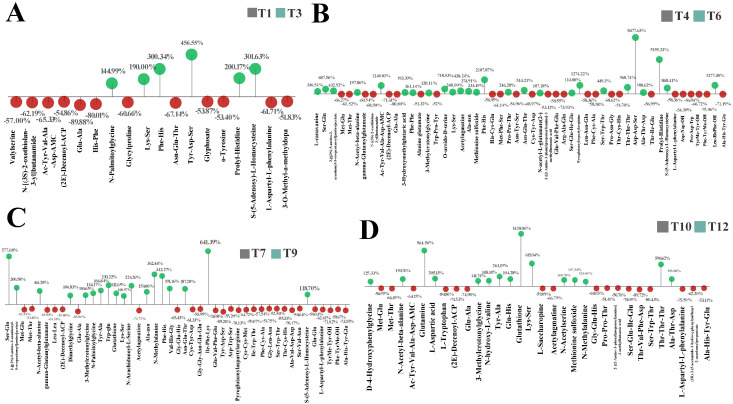
Analysis of amino acid between low (10 mg/L: T1, T4, T7, T10) and high (1000 mg/L: T3, T6, T9, T12) concentration groups. (**A**) T1 vs. T3 (p-hydroxybenzoic acid); (**B**) T4 vs. T6 (salicylic acid); (**C**) T7 vs. T9 (cinnamic acid); (**D**) T10 vs. T12 (ferulic acid). Green: Compared with low-concentration treatments, the contents of amino acid in leaves increased under high-concentration treatments; Red: Compared with low-concentration treatments, the contents of amino acid in leaves decreased under high-concentration treatments.

Compared with low concentrations of phenolic acid (10 mg/L), high concentrations (1000 mg/L) significantly affected phenolic acid and amino acid contents in the leaves (Figure 3 and Figure 4). Under high-concentration treatments, 13 (p-hydroxybenzoic acid), 20 (salicylic acid), 19 (cinnamic acid), and 17 (ferulic acid) differential phenolic acid metabolites were identified (Figure 3). Under high p-hydroxybenzoic acid concentration (1000 mg/L) compared with low concentration (10 mg/L), the contents of cynarine, 2-hydroxybenzaldehyde, 3-methoxyphenol, and 4-methoxysalicylic acid increased by 153.58%, 321.02%, 115.19%, and 145.24%, respectively (Figure 3A). For salicylic acid (T6 vs. T4), 17 phenolic acid metabolites showed significant increases; for example, 2-amino-4-methylphenol increased by 1242.67% (Figure 3B). In addition, under high cinnamic acid concentration (T9 vs. T7), 11 phenolic acid compounds increased significantly (e.g., gallic acid by 641.48%, desaminotyrosine by 534.40%, 4-O-methylgallic acid by 385.12%), while eight decreased (Figure 3C). Under ferulic acid treatment (T12 vs. T10), 17 differential phenolic acids were identified, of which nine increased significantly (e.g., gallic acid by 397.51%, 3,4-dimethylbenzoic acid by 369.17%) (Figure 3D). High concentrations of phenolic acids also resulted in 18 (p-hydroxybenzoic acid), 52 (salicylic acid), 45 (cinnamic acid), and 32 (ferulic acid) differential amino acid metabolites (Figure 4). For p-hydroxybenzoic acid (T3 vs. T1), six amino acid compounds increased significantly (e.g., Tyr-Asp-Ser, 456.55%; S-(5-adenosyl)-L-homocysteine, 301.63%; Asn-Glu-Thr, 300.34%), while 12 decreased (Figure 4A). For salicylic acid (T6 vs. T4), 27 amino acid metabolites increased significantly, with Asp-Asp-Ser increasing by 8477.63% (Figure 4B). Differential compounds such as Ser-Gln (cinnamic acid, +577.68%), Ile-Phe-Lys (cinnamic acid, +641.39%), glutathione (ferulic acid, +1420.06%), and glutamine (ferulic acid, +964.56%) also showed marked increases under high phenolic acid concentrations (Figure 4C,D).

**Figure 5:**
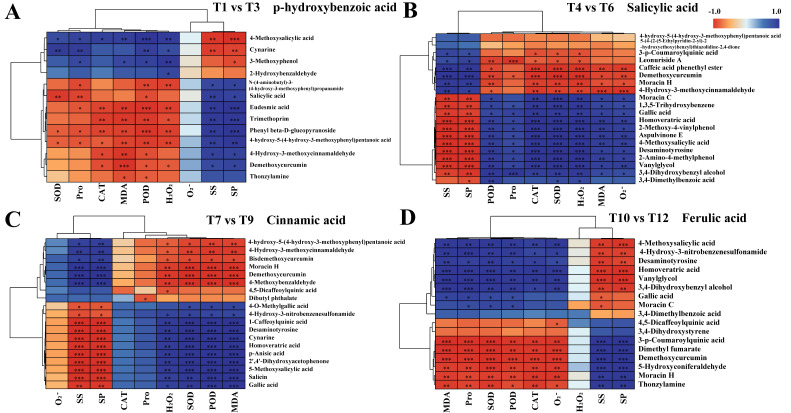
Correlation analysis between plant physiological parameters and phenolic acid under different treatments. (**A**) T1 vs. T3 (p-hydroxybenzoic acid); (**B**) T4 vs. T6 (salicylic acid); (**C**) T7 vs. T9 (cinnamic acid); (**D**) T10 vs. T12 (ferulic acid). *, **, and *** indicate *p* < 0.05, *p* < 0.01, and *p* < 0.001, respectively.

**Figure 6:**
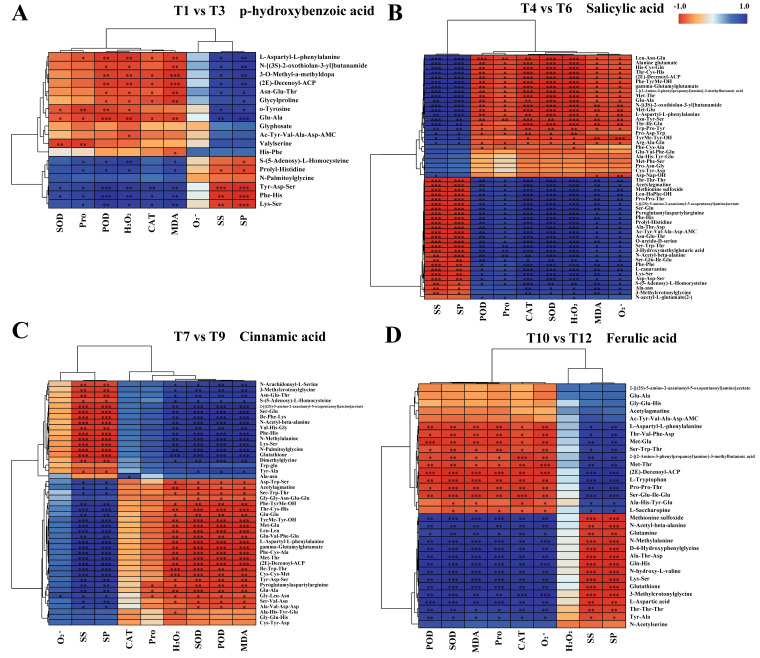
Correlation analysis between plant physiological parameters and amino acids under different treatments. (**A**) T1 vs. T3 (p-hydroxybenzoic acid); (**B**) T4 vs. T6 (salicylic acid); (**C**) T7 vs. T9 (cinnamic acid); (**D**) T10 vs. T12 (ferulic acid). *, **, and *** indicate *p* < 0.05, *p* < 0.01, and *p* < 0.001, respectively.

For p-hydroxybenzoic acid, physiological indicators (Pro, POD, H_2_O_2_, CAT, MDA, SS, and SP) were closely correlated with phenolic acid metabolites (4-methoxysalicylic acid, phenyl β-D-glucopyranoside) and amino acid metabolites (Glu-Ala, Tyr-Asp-Ser, Phe-His) in the leaves (Figure 5A and Figure 6A). Except for certain phenolic acids and amino acids, most differential metabolites exhibited significant correlations with physiological indicators under salicylic acid treatment (Figure 5B and Figure 6B). Similar to p-hydroxybenzoic acid and salicylic acid, differential metabolites in *Coix* leaves showed strong correlations with stress-related physiological indicators under cinnamic acid and ferulic acid treatments (Figure 5C,D and Figure 6C,D). Network analysis further demonstrated close relationships between leaf metabolites under phenolic acid stress and stress-related physiological indicators (Figures S4 and S5).

## 3. Discussion

### 3.1. Effects of Different Treatments on Agronomic Traits of Coix at the Seedling Stage

**Figure 7:**
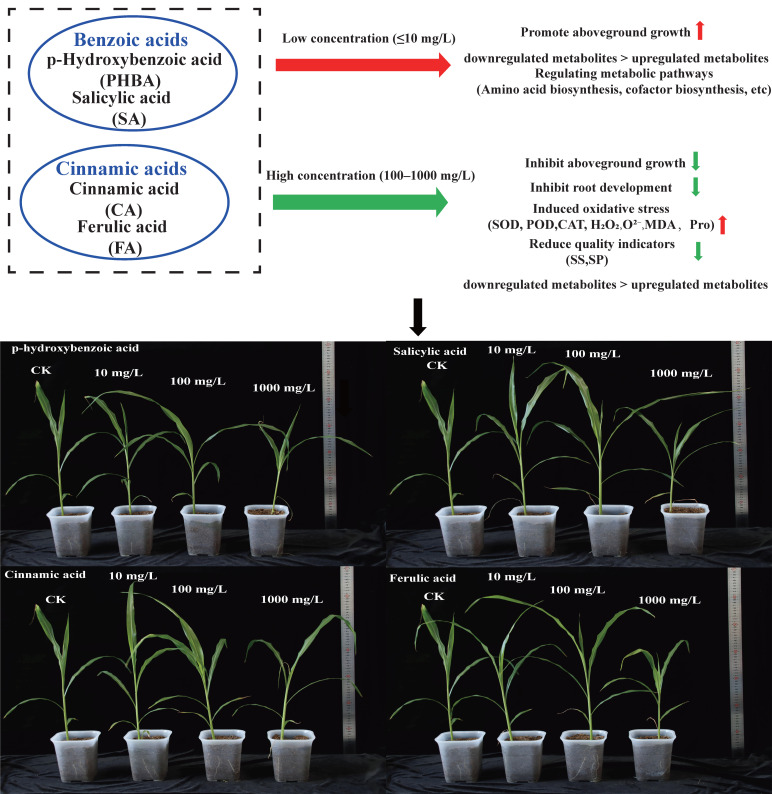
Schematic diagram of the different types and concentrations of phenolic acids on growth, antioxidant defense, and leaf metabolism of *Coix* L. at seedling stage. Red arrows represent ascending. Green arrows represent descending.

Current research indicates that toxic substances in crop root exudates and plant residues are major factors contributing to continuous cropping obstacles, and these toxic substances exist in various forms [20,21]. After rice husks are decomposed by microorganisms, allelochemicals are produced [22]. In our study, different phenolic acids had significant effects on the growth of *Coix* under greenhouse conditions, showing a pattern of growth promotion at low concentrations (≤10 mg/L) and inhibition at high concentrations (≥ 100 mg/L) (Table 1; Figure 7). This may be because low-concentration phenolic compounds act as signaling molecules that promote physiological and metabolic processes, including water uptake, photosynthesis, and respiration [23]. High-concentration phenolic acid stress inhibits plant growth via multiple pathways, including direct damage to plant physiological processes and indirect alterations in soil microbial community composition and enzyme activities [17]. In addition, the development of the *Coix* root system was markedly affected by high phenolic acid concentrations, with root length, root volume, and other root indicators being influenced to varying degrees (Table 2). The inhibitory effects of high phenolic acid concentrations may be attributed to several mechanisms: (1) During germination and seedling stages, phenolic acids influence seed cell division and growth. They inhibit cell elongation and division, ultimately affecting plant development [24]. The results showed that the leaf growth and development of maize seedlings were significantly inhibited under cinnamic acid stress [25]. (2) Allelochemicals disrupt membrane structure, alter membrane permeability, and damage membrane integrity, which can lead to cell death under severe conditions, thereby inhibiting plant growth [26]. Root-secreted allelochemicals can significantly damage the structural integrity of root cell membranes, resulting in increased leakage of intracellular contents [27,28]. (3) The presence of various phenolic acids significantly affected root morphology and the growth of aboveground tissues, leading to changes such as thickened root tips, browning of root caps, and reduced root hair development [29]. Rice root exudates can effectively inhibit the growth and germination of various plants [30]. (4) Toxic substances primarily affect the photosynthetic rate by altering chlorophyll composition and chloroplast structure, ultimately damaging photosynthetic tissues and inhibiting plant growth and development [31,32]. The promoting effects observed at low concentrations of phenolic acids may be attributed to their role as signaling molecules, which enhance physiological and metabolic processes, thereby stimulating plant growth [33]. Moreover, the tolerance index results in our study further confirmed that phenolic acids promoted *Coix* growth at low concentrations while inhibiting growth at high concentrations (Table S1). Different phenolic acids exerted varying degrees of influence on *Coix* growth, with root tissues being more sensitive to allelopathic effects than aerial parts [24]. Specifically, salicylic acid exhibited the strongest growth-promoting effect at 10 mg/L, whereas ferulic acid showed the strongest inhibitory effect at 1000 mg/L (Table 1 and Table 2; Figure 7). This may be related to the selective and specific receptor interactions involved in phenolic acid-mediated allelopathy [34,35].

High concentrations of ferulic acid directly hinder the uptake of mineral ions and water by markedly restraining the net absorption of ions such as NO_3_^−^ and accelerating K^+^ efflux [36]. Exogenous salicylic acid improves the activities of antioxidant enzymes and facilitates glutathione metabolism, thus mitigating damage induced by oxidative stress [37].

### 3.2. Analysis of Antioxidant Enzymes and Antioxidant Substances Under Different Treatments

Numerous studies have shown that the primary cause of continuous cropping obstacles is the allelopathic effects of allelochemicals produced by plants, which inhibit crop growth [38,39,40]. When external environmental stress occurs (high temperature, high salinity, continuous cropping, etc.), the concentration of ROS in plants changes, triggering responses in the internal antioxidant enzyme system and disrupting the redox balance [41]. In our study, with increasing concentration of each phenolic acid, the activities of antioxidant enzymes (SOD, POD, CAT) and the contents of MDA and Pro in *Coix* leaves showed an increasing trend when comparing 1000 mg/L with CK (Table 3; Figure 7). Under external stress, plants regulate themselves to maintain a balance between ROS production and scavenging, thereby sustaining normal cellular metabolic functions [42]. Correlation analysis (Figure 5 and Figure 6) further revealed that pro content was positively correlated with the activities of antioxidant enzymes, indicating that proline collaborates with the enzymatic antioxidant system to mediate defense responses against phenolic acid stress. This result is consistent with previous studies. Under stress conditions, maize maintains osmotic balance by increasing proline content and elevating the activities of antioxidant enzymes to enhance the defense against reactive oxygen species [43]. Several mechanisms may explain this phenomenon: (1) under environmental stress, plants activate antioxidant enzymes and osmotic regulatory substances to eliminate excess ROS and reduce oxidative damage [44]. (2) Environmental stress induces the production of malondialdehyde (MDA), a major product of membrane lipid peroxidation, which damages plant cells [45]. Plants accumulate proline under drought, salt, and low-temperature stresses as it scavenges ROS and stabilizes protein structures [46]. (3) H_2_O_2_ and O_2_^−^ are ROS produced during normal physiological activities. Under environmental stress, these ROS accumulate and cause additional cellular damage [47]^47^. As phenolic acid concentrations increased, soluble sugars and soluble proteins showed a declining trend (Table 3), which are important osmotic regulators that help plants adapt to adverse environmental conditions [48]. As shown in Table 3, antioxidant contents and enzyme activities exhibited significant differences in response to various phenolic acids. Such discrepancies may result from distinct specific signaling pathways induced by different phenolic acid stresses [49,50].

### 3.3. Effects of Different Treatments on Leaf Metabolites of Coix at the Seedling Stage

Abiotic stress typically leads to osmotic and oxidative damage, which in turn affects normal growth, development, and physiological metabolism of plants [51]. In this study, the metabolic processes of *Coix* were significantly affected, with differential metabolites mainly concentrated in organic acids, amino acids, and benzene derivatives after phenolic acid addition (Figure S1 and Figure 1). Free amino acids in plants generally accumulate under stress, and such accumulation has been suggested to correlate with enhanced stress tolerance [52,53]. In this study, under high concentration (1000 mg/L) of each phenolic acid, many amino acid metabolites (e.g., glutathione, aspartic acid, proline) accumulated significantly in the leaves compared with a low concentration (10 mg/L) (Figure 4 and Figure 6). Their accumulation was positively correlated with antioxidant enzyme activities and Pro content, indicating a potential association with stress tolerance (including scavenging ROS and stabilizing protein structures) [46]. As an osmoprotectant, proline helps maintain intracellular water balance under stress conditions [54]. Relevant studies have found that cinnamic acid stress promotes the ASA-GSH cycle in maize leaves, thereby enhancing the scavenging capacity of reactive oxygen species [25]. This result is consistent with recent findings: enhanced biosynthesis of glutathione and flavonoids can activate antioxidant enzymes, thereby alleviating oxidative damage to rice caused by drought stress [55]. In addition to amino acids, which are common osmotic regulators, phenolic compounds (derived from the phenylpropanoid pathway), terpenoids, and other secondary metabolites also play important roles in regulating plant responses to abiotic stress [56,57]. Several phenolic compounds accumulated significantly in our study under high phenolic acid concentrations, suggesting a possible involvement of these metabolites in stress responses [58]. In this study, several phenolic compounds (4-methoxysalicylic acid and gallic acid) accumulated significantly in leaves under high phenolic acid concentrations (Figure 3 and Figure 4). Previous studies have shown that gallic acid promotes plant growth under adverse environmental conditions [59].

## 4. Materials and Methods

### 4.1. Experimental Design

In this study, a pot experiment was conducted in the greenhouse of the Anhui Academy of Agricultural Sciences in Hefei. The temperature was maintained at 25 °C with a relative humidity of 65% throughout the experiment. *Coix lacryma-jobi* L. (*Coix*) served as the research material, with the main cultivar examined being ‘Wanyi 2′, which was provided by the Industrial Crop Institute of the Anhui Academy of Agricultural Sciences and is commonly cultivated in Anhui Province.

A total of 13 treatments with three replicates each were established, involving two factors: the types and concentrations of phenolic acids. Deionized water without phenolic acids was used as a control treatment (CK, 0 mg/L). Four types of phenolic acids were used: p-hydroxybenzoic acid (P), salicylic acid (S), cinnamic acid (C), and ferulic acid (F). Based on the previous germination experiments of *Coix lacryma-jobi* L., four concentrations were set for each phenolic acid: 0 mg/L (CK), 10 mg/L (low), 100 mg/L (middle), and 1000 mg/L (high). All four phenolic acids (≥99%) were purchased from Yuanye Bio-Technology Co., Ltd. (Shanghai, China).

*Coix* seeds were selected and surface-sterilized using 75% ethanol for 30 s, followed by 2% NaClO for 10 min, and then rinsed three times with distilled water for 5 min each. Seeds were evenly sown in substrate soil, with three seeds per pot (12.0 cm in diameter and 15.0 cm in height), filled with 150 g of substrate (peat soil: vermiculite = 1:3) on 9 May 2025. In this study, we used the water-soluble fertilizers “Hua Duo Duo” (20-20-20 + Mg + trace elements). The macronutrient contents of this fertilizer are as follows: total nitrogen (N) 20%, including 5% nitrate nitrogen (NO_3_^−^-N), 5% ammonium nitrogen (NH_4_^+^-N), and 10% urea nitrogen (urea-N); available phosphorus (P_2_O_5_) 20%; available potassium (K_2_O) 20%. The Mg and TE refer to magnesium (0.25%) and trace elements (Fe 0.1% as EDTA-Fe, Mn 0.05%, Zn 0.05%, Cu 0.05%, B 0.02%, Mo 0.005%), respectively. The fertilizer was dissolved in deionized water at 1.0 g/L, applied weekly (500 mL/pot) from seedling emergence until harvest. The first and second applications of phenolic acid treatment (100 mL/pot) were carried out on 27 May 2025 and 4 June 2025, respectively. Then, the seedlings were grown in the substrate until significant differences emerged among treatments, at which point samples were collected on 25 June 2025.

### 4.2. Determination of Agronomic and Physiological Characteristics in Plants

Before harvesting the plants, agronomic traits of *Coix*, including plant height, stem diameter, number of leaves, and SPAD value, were measured. Subsequently, leaves from the different treatments were collected for physiological and non-targeted metabolomic analyses. Three seedlings were randomly selected from each treatment, rinsed with deionized water, and then separated into roots, stems, and leaves using scissors. The fresh weight of each plant part was recorded, and root systems were scanned using a root scanner (Perfection Epson V700 Photo, Seiko Epson Corp., Nagano, Japan). After scanning, total root length, root surface area, root volume, average root diameter, and other root development indicators were calculated using WinRHIZO software (Version 2009, Regent Instruments, Québec City, QC, Canada). Each treatment was performed with three biological replicates (n = 3).

In this study, the physiological indicators measured in the leaves were closely related to stress response, including the activities of superoxide dismutase (SOD), catalase (CAT), and peroxidase (POD), as well as the contents of hydrogen peroxide (H_2_O_2_), superoxide anion (O_2_^−^), malondialdehyde (MDA), proline (Pro), soluble protein (SP), and soluble sugars (SS). Fresh leaf samples (0.5 g) were collected from the third fully expanded leaf from the apex. Details of the measurement methods are as follows: Collect 0.5 g of fresh *Coix lacryma-jobi* L. leaves, which were cut into pieces and placed in a pre-cooled mortar. Then, 5 mL of pre-cooled 50 mmol/L phosphate buffer (pH 7.8) was added, and the mixture was fully ground into a homogenate under ice-bath conditions. The homogenate was transferred to a 10 mL centrifuge tube and centrifuged at 4 °C and 10,000× *g* for 15 min. The resulting supernatant was the crude enzyme extract, which was stored at 4 °C for subsequent activity assays of SOD, POD, and CAT. SOD, POD, and CAT activities were determined using the nitroblue tetrazolium (NBT) photoreduction method, the guaiacol method, and the ammonium molybdate method, respectively [60]. Pro, MDA, SP, and SS contents were measured using the sulfosalicylic acid- ninhydrin method, thiobarbituric acid (TBA) colorimetry, Coomassie Brilliant Blue G-250 colorimetry, and the anthrone-sulfuric acid colorimetric method, respectively [61]. In addition, H_2_O_2_ and O_2_^−^ contents were determined using the titanium sulfate colorimetric method and the hydroxylamine oxidation method, respectively [62]. All agronomic and physiological characteristics were calculated per gram fresh weight (FW) of leaf tissue. Each treatment was performed in 3 biological replicates (n = 3).

### 4.3. Metabolomic Analysis

For metabolomic analysis, five (n = 5) independent biological replicates were set up for each treatment. After four weeks of treatment, UPLC-MS/MS-based methods were used to measure the metabolic characteristics of the leaves, with five biological replicates included in the metabolomics analysis (Maiwei Biotechnology Co., Ltd., Wuhan, China). The specific procedures were as follows: biological samples were placed in a freeze dryer (Scientz-100F, Ningbo Scientz Biotechnology, Ningbo, China) and lyophilized for vacuum freeze-drying for 63 h. The dried samples were ground using a grinder (MM400, Retsch GmbH, Haan, Germany) at 30 Hz for 1.5 min until a fine powder was obtained. The 30 mg of sample powder was weighed and mixed with 1500 μL of a 70% methanol–water extraction solution containing internal standards and pre-cooled to −20 °C. The mixture was vortexed for 30 s every 30 min, for a total of six vortexing cycles. After centrifugation at 12,000 rpm for 3 min, the supernatant was collected, filtered through a 0.22 μm microporous membrane, and stored in sample vials for UPLC-MS/MS analysis. Chromatographic conditions: (1) Column: Waters ACQUITY UPLC HSS T3, 1.8 μm, 2.1 mm × 100 mm. (2) Mobile phase A consisted of ultrapure water with 0.1% formic acid, and mobile phase B was acetonitrile with 0.1% formic acid. (3) The column temperature was set at 40 °C, the flow rate at 0.40 mL/min, and the injection volume at 4 μL. Peaks with a missing rate > 50% in each group of samples were filtered out. Missing values were imputed using the KNN algorithm, and peak areas were corrected via the SVR method. The screened and corrected peaks were used for metabolite identification by searching the in-house database, integrating public databases and prediction libraries, and applying the metDNA method. Differential metabolites (DMs) were identified using the criteria VIP > 1 and *p* < 0.05.

### 4.4. Data Analysis

In this study, R software (version 4.1.1, Auckland, New Zealand) was used to perform one-way ANOVA or two-way ANOVA to evaluate differences among treatments, followed by Duncan’s test (*p* < 0.05). Two-way ANOVA was performed only on the 12 treatment combinations (4 types × 3 concentrations) to evaluate the main effects and interaction of type and concentration. A one-way analysis of variance (ANOVA) was subsequently conducted on all treatments, including the control group (CK), to compare the differences among various concentrations and types of phenolic acids. MetaboAnalyst was used for pathway enrichment analysis for DEMs. The metabolites identified in metabolomics were mapped to KEGG pathway database for biological function interpretation. Visualization of metabolomics data was performed using the Maiwei Cloud Platform (https://cloud.metware.cn/).

## 5. Conclusions

Our results demonstrate that the type and concentration of phenolic acids affect the growth and development of *Coix* under greenhouse conditions. Based on agronomic traits and tolerance coefficients, we found that phenolic acid concentrations ≤10 mg/L promote the growth of *Coix*, whereas concentrations ≥100 mg/L inhibit crop growth. In addition, the response of *Coix* to different phenolic acids varied significantly. At high concentrations (≥100 mg/L), ferulic acid exhibited the strongest inhibitory effect on *Coix* growth, while at low concentrations (≤10 mg/L), salicylic acid showed the greatest promoting effect. Furthermore, compared with the CK, the activities of antioxidant enzymes (POD, CAT, SOD) and the contents of antioxidant-related substances (H_2_O_2_, O_2_^−^, MDA, Pro) in *Coix* leaves increased significantly under high concentration (1000 mg/L), whereas SS and SP decreased markedly. Phenolic acids also altered the composition of leaf metabolites, with most differential metabolites showing a decreasing trend after phenolic acid addition. Compared with low phenolic acid concentrations (≤10 mg/L), differential metabolites under high concentrations (≥100 mg/L) were mainly associated with amino acid and phenolic acid metabolic pathways. Specifically, under high concentration (1000 mg/L) compared with low concentration (10 mg/L), the accumulation of specific amino acids (e.g., proline, glutathione) and phenolic metabolites (e.g., gallic acid, 4-methoxysalicylic acid) reflects adaptive metabolic adjustments under phenolic acid stress. Our study indicates that phenolic acids can induce stress responses and growth inhibition in *Coix* seedlings, providing a basis for future field studies on continuous cropping obstacles. From an agronomic management perspective, strategies to mitigate continuous cropping obstacles in *Coix* should focus on: (i) reducing the accumulation of highly inhibitory phenolic acids (particularly ferulic acid) through crop rotation with species that degrade or transform these compounds, or through the application of biochar or microbial inoculants known to metabolize phenolic allelochemicals; (ii) potentially leveraging low concentrations of salicylic acid as a cost-effective seed or seedling treatment to improve stress resilience. Nevertheless, future research should integrate metabolomic analysis of root exudates with long-term field monitoring to better clarify the mechanism of phenolic acid-induced continuous cropping obstacles in *Coix*.

## Figures and Tables

**Table 1:** Effects of different types and concentrations of phenolic acids on agronomic traits of *Coix* L. at seedling stage.

Treatment		Fresh Biomass			Dry Biomass			Plant Height/cm	Stem Diameter/mm	SPAD	Leaf Number
		Leaf/g	Stem/g	Root/g	Leaf/g	Stem/g	Root/g				
p-hydroxybenzoic acid	CK (0 mg/L)	10.46 ± 0.59 b	23.74 ± 2.22 a	13.03 ± 0.09 b	1.40 ± 0.17 a	1.21 ± 0.21 b	0.68 ± 0.04 b	116.12 ± 4.76 a	10.12 ± 0.39 a	41.80 ± 1.50 ab	7.33 ± 0.58 a
	T1(10 mg/L)	11.61 ± 1.38 abAB	28.38 ± 2.08 aA	17.95 ± 1.89 aAB	1.54 ± 0.29 aAB	1.92 ± 0.21 aA	1.11 ± 0.10 aAB	118.33 ± 6.45 aB	8.89 ± 0.37 bA	43.93 ± 0.85 aA	7.33 ± 0.58 aAB
	T2(100 mg/L)	13.53 ± 1.75 aA	25.51 ± 4.76 aAB	11.89 ± 2.23 bAB	1.70 ± 0.34 aA	1.53 ± 0.44 abAB	0.60 ± 0.04 bB	120.03 ± 7.86 aAB	10.76 ± 0.93 aA	44.07 ± 4.54 aA	7.67 ± 0.58 aA
	T3(1000 mg/L)	4.53 ± 0.45 cAB	7.01 ± 0.75 bB	5.12 ± 0.40 cB	0.52 ± 0.02 bA	0.34 ± 0.03 cC	0.34 ± 0.04 cB	69.00 ± 2.46 bC	7.70 ± 0.10 cB	38.43 ± 1.10 bA	6.67 ± 0.58 aA
Salicylic acid	CK (0 mg/L)	10.46 ± 0.59 b	23.74 ± 2.22 c	13.03 ± 0.09 b	1.40 ± 0.17 a	1.21 ± 0.21 c	0.68 ± 0.04 c	116.12 ± 4.76 b	10.12 ± 0.39 a	41.80 ± 1.50 a	7.33 ± 0.58 a
	T4(10 mg/L)	12.40 ± 1.64 aA	36.94 ± 4.38 aA	20.88 ± 1.94 aA	1.66 ± 0.27 aA	2.64 ± 0.54 aA	1.17 ± 0.12 aA	140.40 ± 1.83 aA	10.67 ± 2.55 aA	43.67 ± 1.50 aA	7.00 ± 0.01 abB
	T5(100 mg/L)	11.69 ± 0.80 abA	29.15 ± 1.06 bA	14.59 ± 1.44 bA	1.52 ± 0.19 aA	1.90 ± 0.16 bA	0.96 ± 0.08 bA	131.23 ± 4.49 aA	10.45 ± 1.38 aA	32.10 ± 1.39 cB	6.33 ± 0.58 bB
	T6(1000 mg/L)	4.09 ± 0.44 cB	7.86 ± 1.34 dB	5.73 ± 0.76 cB	0.41 ± 0.05 bA	0.47 ± 0.04 dB	0.36 ± 0.07 dB	81.87 ± 7.10 cB	5.96 ± 0.22 bA	36.43 ± 0.81 bB	7.00 ± 0.01 abA
Cinnamic acid	CK (0 mg/L)	10.46 ± 0.59 a	23.74 ± 2.22 b	13.03 ± 0.09 ab	1.40 ± 0.17 a	1.21 ± 0.21 b	0.68 ± 0.04 b	116.12 ± 4.76 b	10.12 ± 0.39 b	41.80 ± 1.50 a	7.33 ± 0.58 a
	T7(10 mg/L)	11.71 ± 1.82 aAB	37.91 ± 4.53 aA	16.35 ± 3.81 aAB	1.58 ± 0.27 aAB	1.73 ± 0.41 aA	0.98 ± 0.09 aAB	138.00 ± 6.62 aA	10.99 ± 0.70 aA	37.43 ± 2.44 bcB	7.67 ± 0.58 aAB
	T8(100 mg/L)	8.06 ± 0.14 bB	18.21 ± 0.92 cBC	9.01 ± 0.87 cB	1.05 ± 0.03 bB	1.05 ± 0.03 bB	0.51 ± 0.05 cB	113.30 ± 5.07 bB	9.14 ± 0.12 cA	35.47 ± 2.16 cB	6.00 ± 0.01 bB
	T9(1000 mg/L)	5.13 ± 0.55 cA	15.38 ± 0.60 cA	10.61 ± 0.53 bcA	0.51 ± 0.05 cA	0.58 ± 0.04 cA	0.52 ± 0.05 cA	93.00 ± 0.62 cA	8.40 ± 0.27 cB	40.67 ± 0.40 abA	7.00 ± 0.01 aA
Ferulic acid	CK (0 mg/L)	10.46 ± 0.59 a	23.74 ± 2.22 b	13.03 ± 0.09 ab	1.40 ± 0.17 a	1.21 ± 0.21 b	0.68 ± 0.04 b	116.12 ± 4.76 b	10.12 ± 0.39 a	41.80 ± 1.50 a	7.33 ± 0.58 ab
	T10(10 mg/L)	9.32 ± 0.66 bB	35.25 ± 7.04 aA	14.49 ± 1.97 aB	1.12 ± 0.21 abB	2.55 ± 0.56 aA	0.90 ± 0.16 aB	140.60 ± 8.61 aA	10.64 ± 2.20 aA	39.13 ± 2.47 aB	8.00 ± 0.01 aA
	T11(100 mg/L)	7.9 ± 0.76 cB	23.37 ± 2.33 bC	10.48 ± 2.37 bB	0.90 ± 0.09 bB	1.22 ± 0.26 bB	0.36 ± 0.08 cC	114.77 ± 6.35 bB	9.05 ± 1.07 abA	43.20 ± 0.66 aA	7.33 ± 0.58 abA
	T12(1000 mg/L)	4.35 ± 0.25 dAB	6.59 ± 0.17 cB	5.86 ± 0.32 cB	0.29 ± 0.09 cB	0.28 ± 0.07 cC	0.35 ± 0.03 cB	68.20 ± 1.82 cC	7.54 ± 0.50 bB	35.13 ± 2.94 bB	7.00 ± 0.01 bA
C(Concentration)		142.53 **	185.61 **	100.53 **	98.51 **	103.67 **	182.51 **	212.30 **	6.77 **	13.13 **	10.43 **
T(Type)		11.64 **	3.35 *	5.43 **	11.27 **	5.23 **	18.22 **	61.35 **	2.71	13.39 **	7.00 **
C*T		7.22 **	6.39 **	5.97 **	2.93 *	3.19 *	9.23 **	25.15 **	2.1	12.80 **	3.57 *

Note: Lowercase letters indicate significant differences among different concentrations of phenolic acids according to Duncan’s test (*p* < 0.05). Capital letters indicate significant differences among different types of phenolic acids according to Duncan’s test (*p* < 0.05). Values are presented as means ± SD (n = 3). The results of two-way analysis of variance are presented as F value and *p* value. * *p* < 0.05; ** *p* < 0.01.

**Table 2:** Effects of different types and concentrations of phenolic acids on root development of *Coix* L. at seedling stage.

Treatment		Length (cm)	Surface Area (cm^2^)	Average Diameter (mm)	Root Volume (cm^3^)	Tips
p-hydroxybenzoic acid	CK (0 mg/L)	2097.82 ± 389.08 b	312.33 ± 45.32 b	0.48 ± 0.02 a	3.83 ± 0.22 b	12,865.67 ± 2347.48 b
	T1(10 mg/L)	3372.82 ± 378.59 aA	476.61 ± 62.01 aAB	0.45 ± 0.03 aA	5.37 ± 0.88 aA	32,177.00 ± 2305.37 aB
	T2(100 mg/L)	2082.51 ± 138.86 bB	300.05 ± 35.93 bB	0.47 ± 0.01 aA	3.45 ± 0.60 bB	16,585.67 ± 1478.44 bC
	T3(1000 mg/L)	1560.31 ± 344.23 bB	187.76 ± 39.24 cB	0.31 ± 0.01 bB	1.70 ± 0.08 cC	15,196.67 ± 2088.65 bA
Salicylic acid	CK (0 mg/L)	2097.82 ± 389.08 b	312.33 ± 45.32 c	0.48 ± 0.02 b	3.83 ± 0.22 c	12,865.67 ± 2347.48 b
	T4(10 mg/L)	3789.53 ± 406.45 aA	548.82 ± 38.81 aA	0.45 ± 0.02 aA	6.23 ± 0.46 aA	33,512.00 ± 4249.41 aB
	T5(100 mg/L)	3250.25 ± 85.73 aA	443.79 ± 16.36 bA	0.44 ± 0.01 bB	4.82 ± 0.23 bA	37,560.33 ± 1188.53 aB
	T6(1000 mg/L)	2033.70 ± 562.45 bB	264.94 ± 38.39 cAB	0.32 ± 0.01 cB	2.41 ± 0.24 dB	29,378.33 ± 8883.33 aA
Cinnamic acid	CK (0 mg/L)	2097.82 ± 389.08 c	312.33 ± 45.32 b	0.48 ± 0.02 b	3.83 ± 0.22 b	12,865.67 ± 2347.48 c
	T7(10 mg/L)	3469.53 ± 423.60 abA	478.41 ± 69.58 aAB	0.44 ± 0.05 aA	5.72 ± 0.44 aA	44,370.00 ± 3434.83 aA
	T8(100 mg/L)	3673.12 ± 210.88 aA	383.44 ± 10.77 abA	0.34 ± 0.01 aC	3.31 ± 0.32 bB	47,232.67 ± 2740.57 aA
	T9(1000 mg/L)	2930.22 ± 379.19 bA	317.00 ± 84.81 bA	0.36 ± 0.03 bA	3.30 ± 0.66 bA	25,386.67 ± 11,509.33 bA
Ferulic acid	CK (0 mg/L)	2097.82 ± 389.08 a	312.33 ± 45.32 a	0.48 ± 0.02 a	3.83 ± 0.22 ab	12,865.67 ± 2347.48 a
	T10(10 mg/L)	2343.83 ± 358.79 aB	341.00 ± 104.82 aB	0.48 ± 0.07 aA	4.72 ± 1.12 aA	16,475.67 ± 2661.94 aC
	T11(100 mg/L)	1889.48 ± 468.99 aB	259.05 ± 73.89 aB	0.43 ± 0.02 aB	3.13 ± 0.53 bcB	14,558.00 ± 2609.84 aC
	T12(1000 mg/L)	2075.31 ± 81.88 aB	220.06 ± 12.88 aAB	0.36 ± 0.01 bA	2.06 ± 0.12 cBC	15,602.67 ± 331.63 aA
C(Concentration)		28.88 **	42.60 **	55.43 **	96.24 **	14.75 **
T(Type)		24.71 **	12.38 **	4.14 *	8.60 **	45.51 **
C*T		5.47 **	1.99	5.85 **	2.80 *	5.85 **

Note: Lowercase letters indicate significant differences among different concentrations of phenolic acids according to Duncan’s test (*p* < 0.05). Capital letters indicate significant differences among different types of phenolic acids according to Duncan’s test (*p* < 0.05). Values are presented as means ± SD (n = 3). The results of two-way analysis of variance are presented as F value and *p* value. * *p* < 0.05; ** *p* < 0.01.

**Table 3:** Effects of different types and concentrations of phenolic acids on physiological parameters of *Coix L*. at seedling stage.

Treatment		SOD (U·g^−1^ FW)	POD (U·g^−1^ FW)	CAT (mmolh^−1^·g^−1^ FW)	H_2_O_2_ (μmol·g^−1^ FW)	O_2_^−^(nmol·g^−1^ FW)	MDA (nmol·g^−1^ FW)	Pro (μg·g^−1^ FW)	SS (mg·g^−1^ FW)	SP (mg·g^−1^ FW)
p-hydroxybenzoic acid	CK (0 mg/L)	227.42 ± 3.92 b	249.29 ± 7.86 c	0.41 ± 0.05 c	7.53 ± 0.32 b	30.14 ± 0.86 a	4.99 ± 0.21 c	3.43 ± 0.14 b	5.39 ± 0.18 b	1.21 ± 0.05 a
	T1 (10 mg/L)	229.16 ± 8.58 bC	235.63 ± 3.83 cB	0.48 ± 0.04 cA	7.71 ± 0.29 bB	28.12 ± 1.36 aB	5.22 ± 0.52 cB	3.73 ± 0.49 bA	6.55 ± 0.31 aC	1.10 ± 0.03 bA
	T2 (100 mg/L)	234.51 ± 5.49 abB	299.11 ± 8.49 bA	0.64 ± 0.02 bA	7.79 ± 0.08 bA	27.61 ± 5.33 aB	5.95 ± 0.20 bB	4.46 ± 0.13 aA	5.19 ± 0.15 bcC	0.92 ± 0.04 cA
	T3 (1000 mg/L)	244.22 ± 3.21 aC	322.33 ± 11.54 aA	0.76 ± 0.10 aA	9.01 ± 0.25 aA	28.03 ± 0.98 aC	6.88 ± 0.10 aB	4.90 ± 0.19 aA	4.83 ± 0.20 cC	0.44 ± 0.02 dD
Salicylic acid	CK (0 mg/L)	227.42 ± 3.92 d	249.29 ± 7.86 c	0.41 ± 0.05 c	7.53 ± 0.32 b	30.14 ± 0.86 b	4.99 ± 0.21 b	3.43 ± 0.14 c	5.39 ± 0.18 c	1.21 ± 0.05 a
	T4 (10 mg/L)	281.22 ± 4.33 cA	253.44 ± 8.24 bcA	0.43 ± 0.02 cB	7.20 ± 0.08 bB	32.38 ± 4.55 bAB	5.49 ± 0.31 bB	4.14 ± 0.54 bcA	9.94 ± 0.24 aA	0.90 ± 0.03 bB
	T5 (100 mg/L)	312.68 ± 8.65 bAB	267.41 ± 7.49 bB	0.57 ± 0.01 bB	7.55 ± 0.20 bA	35.59 ± 5.10 bA	5.39 ± 0.24 bC	4.65 ± 0.53 abA	6.59 ± 0.31 bA	0.87 ± 0.03 bB
	T6 (1000 mg/L)	532.55 ± 10.70 aA	292.04 ± 8.41 aB	0.84 ± 0.04 aA	9.43 ± 0.08 aA	44.18 ± 0.85 aB	6.61 ± 0.26 aB	5.36 ± 0.16 aA	5.68 ± 0.20 cA	0.58 ± 0.03 cB
Cinnamic acid	CK (0 mg/L)	227.42 ± 3.92 b	249.29 ± 7.86 c	0.41 ± 0.05 a	7.53 ± 0.32 c	30.14 ± 0.86 a	4.99 ± 0.21 b	3.43 ± 0.14 c	5.39 ± 0.18 c	1.21 ± 0.05 a
	T7 (10 mg/L)	218.47 ± 1.20 cC	199.57 ± 4.69 dC	0.35 ± 0.01 bcC	8.04 ± 0.25 bB	31.11 ± 1.38 aB	5.00 ± 0.05 bB	3.64 ± 0.58 bcA	8.50 ± 0.18 aB	0.93 ± 0.03 bB
	T8 (100 mg/L)	221.69 ± 5.85 bcB	300.50 ± 8.13 bA	0.31 ± 0.01 cC	8.29 ± 0.35 bA	24.98 ± 0.88 bB	5.18 ± 0.03 bC	4.48 ± 0.09 abA	5.82 ± 0.09 bB	0.55 ± 0.01 cD
	T9 (1000 mg/L)	254.08 ± 1.80 aC	322.92 ± 11.73 aA	0.37 ± 0.02 abC	9.18 ± 0.10 aA	29.12 ± 2.12 aC	9.92 ± 0.39 aA	4.78 ± 0.70 aA	5.56 ± 0.05 bcAB	0.49 ± 0.01 cC
Ferulic acid	CK (0 mg/L)	227.42 ± 3.92 c	249.29 ± 7.86 b	0.41 ± 0.05 c	7.53 ± 0.32 a	30.14 ± 0.86 c	4.99 ± 0.21 c	3.43 ± 0.14 b	5.39 ± 0.18 b	1.21 ± 0.05 a
	T10 (10 mg/L)	250.37 ± 7.67 bB	225.43 ± 9.38 cB	0.44 ± 0.02 cAB	8.52 ± 0.25 aA	36.20 ± 1.24 bA	6.85 ± 0.25 bA	3.72 ± 0.10 bA	8.68 ± 0.41 aB	1.11 ± 0.02 bA
	T11 (100 mg/L)	366.50 ± 8.37 aA	252.82 ± 4.10 bC	0.67 ± 0.03 aA	8.32 ± 1.01 aA	38.16 ± 1.47 bA	7.08 ± 0.22 bA	4.41 ± 0.34 aA	5.54 ± 0.01 bB	0.72 ± 0.02 cC
	T12 (1000 mg/L)	379.34 ± 8.76 aB	269.47 ± 3.50 aC	0.55 ± 0.02 bB	8.48 ± 0.48 aB	50.50 ± 2.36 aA	9.54 ± 0.38 aA	4.77 ± 0.17 aA	5.31 ± 0.21 bB	0.76 ± 0.02 cA
C(Concentration)		755.88 **	268.64 **	91.61 **	34.03 **	19.38 **	308.42 **	25.46 **	656.35 **	958.91 **
T(Type)		976.34 **	33.14 **	110.31 **	2.85 **	52.11 **	91.67 **	2.30	106.10 **	116.00 **
C*T		261.25 **	24.72 **	25.41 **	5.08 **	7.06 **	37.19 **	0.18	19.85 **	86.40 **

Note: Lowercase letters indicate significant differences among different concentrations of phenolic acids according to Duncan’s test (*p* < 0.05). Capital letters indicate significant differences among different types of phenolic acids according to Duncan’s test (*p* < 0.05). Values are presented as means ± SD (n = 3). The results of two-way analysis of variance are presented as F value and *p* value. * *p* < 0.05; ** *p* < 0.01. POD (peroxidase), CAT (catalase), SOD (superoxide dismutase), H_2_O_2_ (hydrogen peroxide), O_2_^−^ (superoxide anion), MDA (malondialdehyde), Pro (proline), SS (soluble sugars), SP (soluble protein).

## Data Availability

All data generated or analyzed during this study are included within this published article and its Supplementary Information Files.

## Supplementary Materials

The following supporting information can be downloaded at: https://www.mdpi.com/article/10.3390/plants15132015/s1, Figure S1: The scatter diagram of differential substance classification between experimental groups (10 mg/L and 1000 mg/L) and control groups (CK); Figure S2: The Sankey and bubble plot of KEGG (top 10 pathway) between experimental groups (10 mg/L and 1000 mg/L) and control groups (CK); Figure S3: The dynamic distribution map of differences in metabolite between experimental groups (10 mg/L and 1000 mg/L) and control groups (CK); Figure S4: The network analysis between plant physiological and phenolic acid under different treatments; Figure S5: The network analysis between plant physiological and amino acid under different treatments; Table S1: Effects of different types and concentrations of phenolic acids on tolerance index and root structure of *Coix*. L at seedling stage.
