# Supplementary material for: Responses of Coix lacryma-jobi L. to Exogenous Phenolic Acid Treatments: Effects on Growth, Antioxidant Responses, and Leaf Metabolome
Source: Plants (Basel). 2026 Jun 29;15(13):2015. doi: 10.3390/plants15132015 (PMC13364040; doi:10.3390/plants15132015)
Supplement: Supplementary file 1 [file plants-15-02015-s001.zip › plants-4358447-supplementary.pdf]

Table S1 Effects of different types and concentrations of phenolic acids on tolerance index and root structure of *Coix* L. at seedling stage

Treatment		TIA	TIU	RLR	RF	RMR	RTD
p-hydroxybenzoic acid	CK (0 mg/L)			3105.76±520.23b	548.90±103.23b	0.21±0.03b	0.18±0.01a
	T1(10 mg/L)	1.35±0.34aA	1.64±0.08aA	3049.78±216.76bAB	634.31±71.98bA	0.24±0.02abA	0.21±0.02aA
	T2(100 mg/L)	1.25±0.21aA	0.89±0.12bB	3491.42±280.33abC	611.91±68.29bB	0.16±0.01cBC	0.18±0.03aA
	T3(1000 mg/L)	0.34±0.06bAB	0.50±0.06cB	4687.09±1256.23aA	916.2±184.99aA	0.28±0.03aB	0.20±0.02aA
Salicylic acid	CK (0 mg/L)			3105.76±520.23b	548.90±103.23b	0.21±0.03b	0.18±0.01a
	T4(10 mg/L)	1.66±0.35aA	1.75±0.29aA	3225.53±93.04bAB	608.29±40.58bA	0.22±0.01bAB	0.19±0.02aAB
	T5(100 mg/L)	1.32±0.19aA	1.43±0.12aA	3396.34±341.92bC	674.37±16.90abB	0.22±0.02bA	0.20±0.02aA
	T6(1000 mg/L)	0.34±0.05bAB	0.53±0.08bB	5693.27±1354.74aA	836.55±180.46aA	0.29±0.03aB	0.15±0.01bB
Cinnamic acid	CK (0 mg/L)			3105.76±520.23c	548.90±103.23c	0.21±0.03b	0.18±0.01a
	T7(10 mg/L)	1.26±0.15aA	1.46±0.23aA	3543.37±327.94cA	606.60±56.38cA	0.23±0.02bAB	0.17±0.01aB
	T8(100 mg/L)	0.81±0.11bB	0.75±0.08bBC	7279.46±845.17aA	1116.83±130.21aA	0.19±0.01bAB	0.16±0.03aAB
	T9(1000 mg/L)	0.42±0.08cA	0.77±0.12bA	5647.01±237.55bA	896.87±85.10bA	0.32±0.02aAB	0.16±0.02aB
Ferulic acid	CK (0 mg/L)			3105.76±520.23b	548.90±103.23b	0.21±0.03b	0.18±0.01a
	T10(10 mg/L)	1.42±0.22aA	1.34±0.34aA	2656.28±577.59bB	508.22±108.60bA	0.20±0.01bB	0.19±0.01aAB
	T11(100 mg/L)	0.82±0.14bB	0.54±0.14bC	5198.28±420.45aB	600.43±81.36bB	0.15±0.03bC	0.12±0.02bB
	T12(1000 mg/L)	0.22±0.09cB	0.52±0.05bB	5970.93±546.96aA	1009.91±102.9aA	0.39±0.07aA	0.17±0.01aAB
C(Concentration)		99.074**	102.994**	40.665**	28.271**	70.755**	7.662**
T(Type)		4.533*	10.019**	11.572**	5.308**	0.948	7.089**
C*T		2.219	5.150**	6.920**	6.526**	6.535**	5.166**

Note: Lowercase letters indicate significant differences among different concentrations of phenolic acids according to Duncan test ($P < 0.05$). Capital letters indicate significant differences among different types of phenolic acids according to Duncan test ($P < 0.05$). Values are presented as means ± SD (n = 3). The results of two-way analysis of variance are presented as F value and p value. * $P < 0.05$; ** $P < 0.01$. The TIA, TIU, RLR, RF, RMR, and RTD indicate tolerance index of aboveground, tolerance index of underground, root length ratio, root fineness, root mass ratio, and root tissue density.

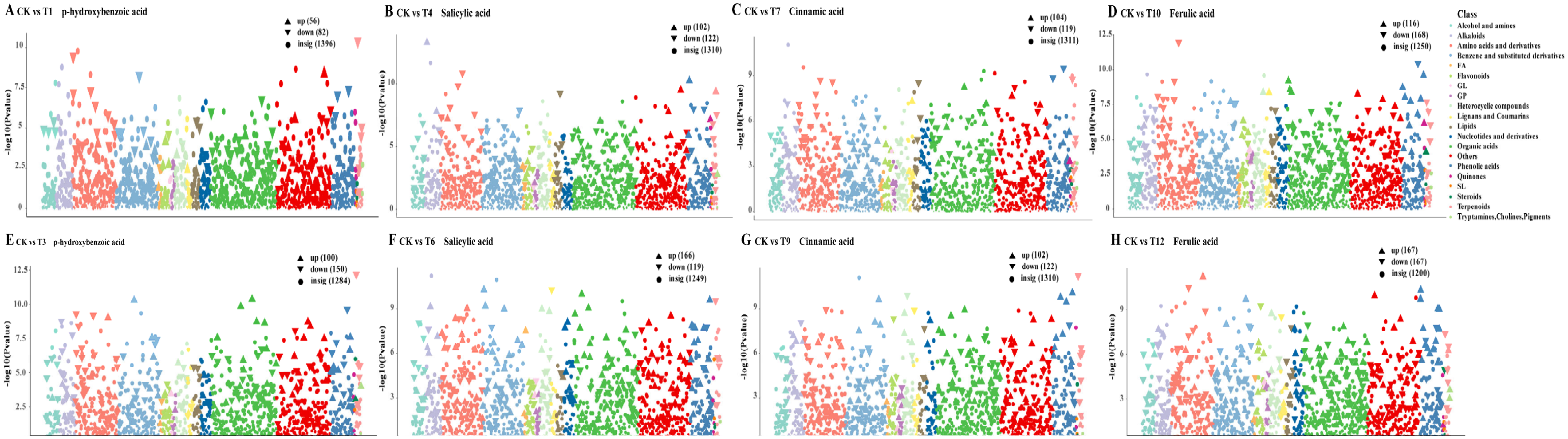

Figure S1. The scatter diagram of differential substance classification between experimental groups (10 mg/L and 1000 mg/L) and control groups (CK). Low concentration: T1 (p-hydroxybenzoic acid), T4 (salicylic acid), T7 (cinnamic acid), T10 (ferulic acid); High concentration: T3 (p-hydroxybenzoic acid), T6 (salicylic acid), T9 (cinnamic acid), T12 (ferulic acid).

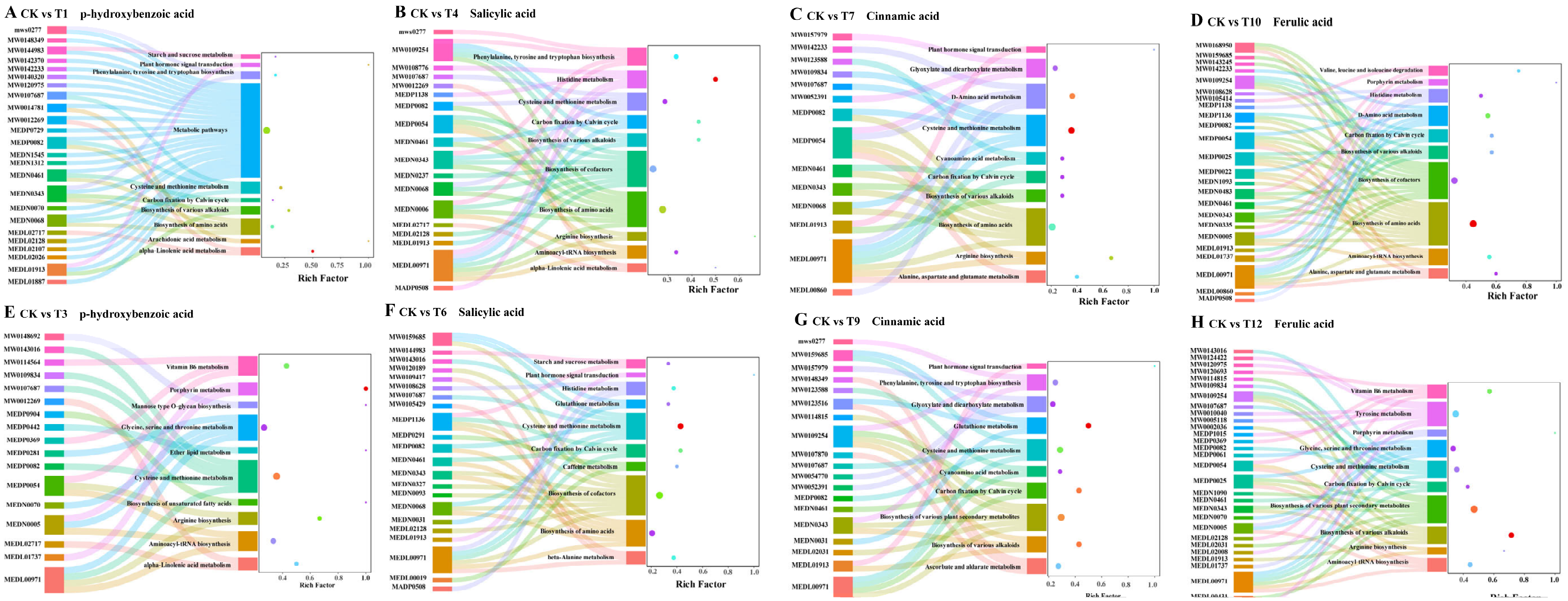

Figure S2. The Sankey and bubble plot of KEGG (top 10 pathway) between experimental groups (10 mg/L and 1000 mg/L) and control groups (CK). Low concentration: T1 (p-hydroxybenzoic acid), T4 (salicylic acid), T7 (cinnamic acid), T10 (ferulic acid); High concentration: T3 (p-hydroxybenzoic acid), T6 (salicylic acid), T9 (cinnamic acid), T12 (ferulic acid).

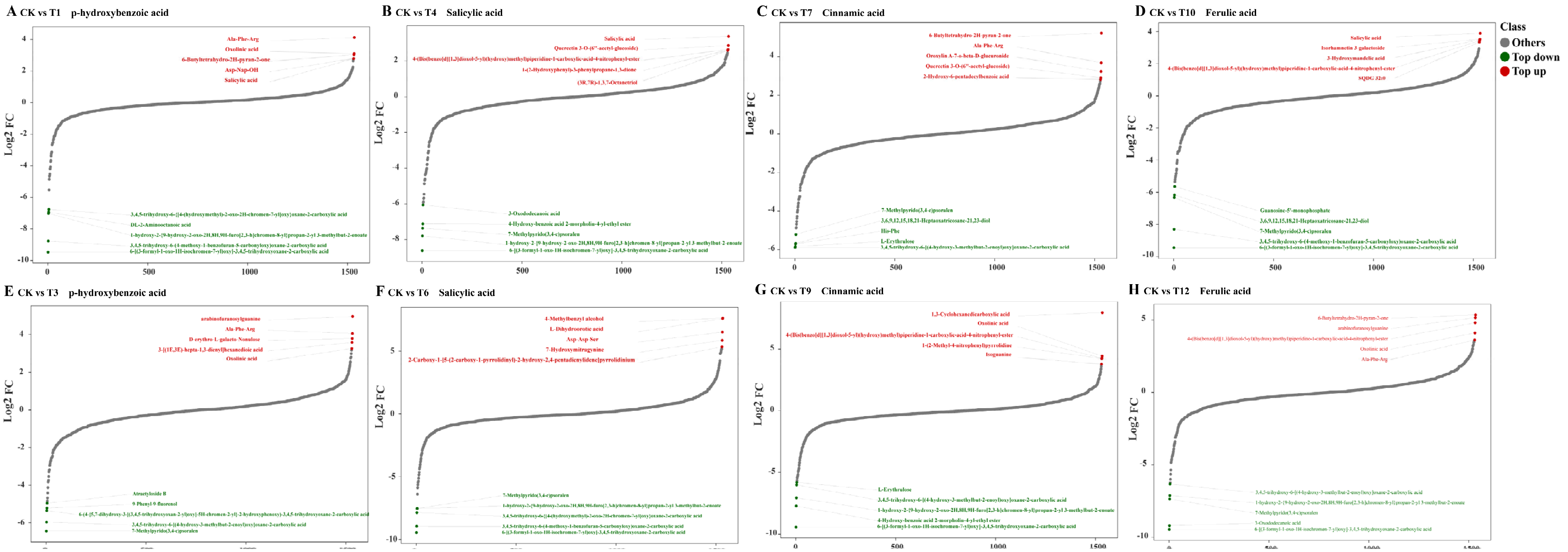

Figure S3. The dynamic distribution map of differences in metabolite between experimental groups (10 mg/L and 1000 mg/L) and control groups (CK). Low concentration: T1 (p-hydroxybenzoic acid), T4 (salicylic acid), T7 (cinnamic acid), T10 (ferulic acid); High concentration: T3 (p-hydroxybenzoic acid), T6 (salicylic acid), T9 (cinnamic acid), T12 (ferulic acid). Each point represent one metabolite, red represent top 5 increased metabolites, green represent top 5 decreased metabolites.

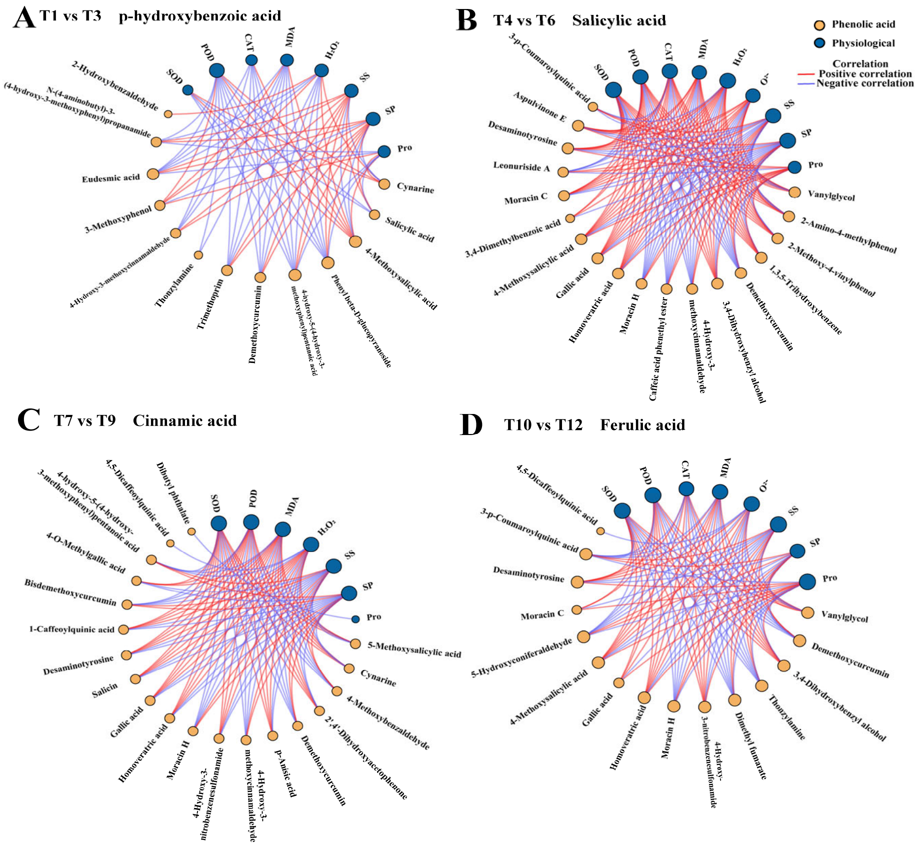

Figure S4. The network analysis between plant physiological and phenolic acid under different treatments. A, B, C and D represent T1 vs T3 (p-hydroxybenzoic acid), T4 vs T6 (salicylic acid), T7 vs T9 (cinnamic acid), and T10 vs T12 (ferulic acid), respectively. Colors indicate correlation type: purple for negative correlations, red for positive correlations.

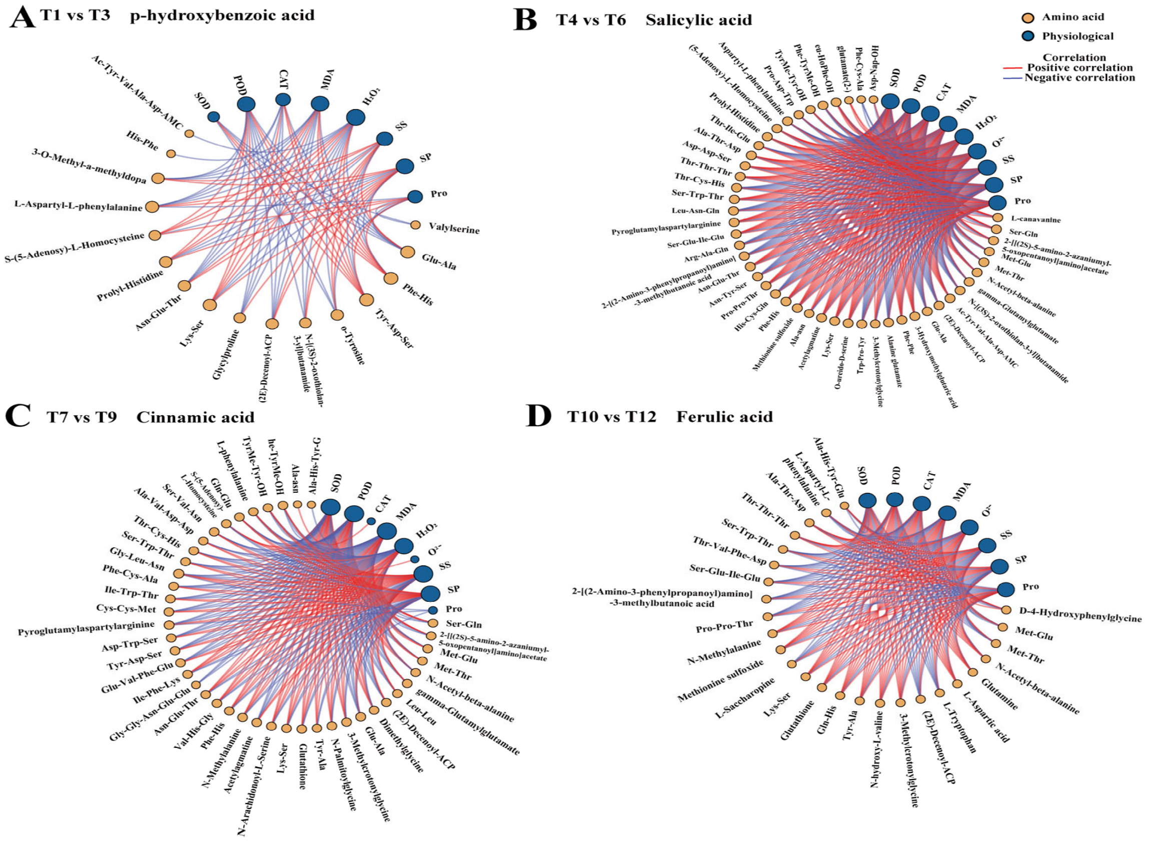

Figure S5. The network analysis between plant physiological and amino acid under different treatments. A, B, C and D represent T1 vs T3 (p-hydroxybenzoic acid), T4 vs T6 (salicylic acid), T7 vs T9 (cinnamic acid), and T10 vs T12 (ferulic acid), respectively. Colors indicate correlation type: purple for negative correlations, red for positive correlations.
